# Supplementary material for: Causal Relationship between Aging and Anorexia Nervosa: A White-Matter-Microstructure-Mediated Mendelian Randomization Analysis
Source: Biomedicines. 2024 Aug 16;12(8):1874. doi: 10.3390/biomedicines12081874 (PMC11351342; doi:10.3390/biomedicines12081874)
Supplement: Supplementary file 1 [file biomedicines-12-01874-s001.zip › Supplementary Figure/Figure S4/Singe SNP plot of MR analysis for TL to ubm-b-2024.pdf]

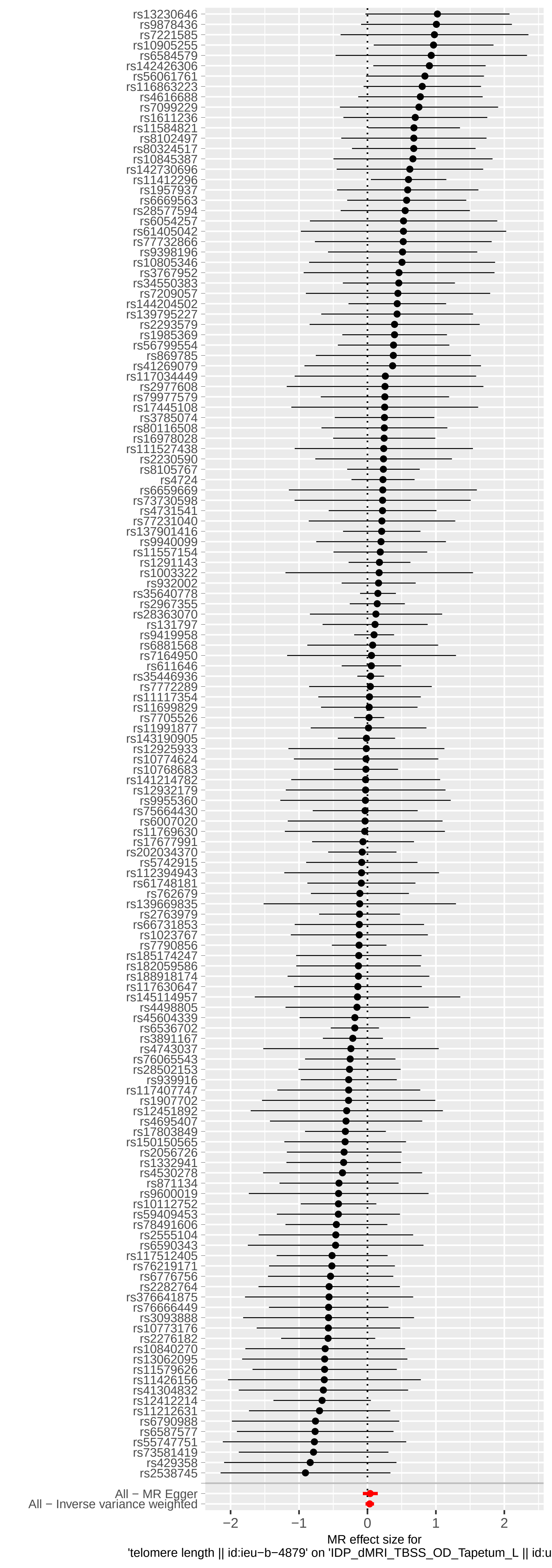
rs13230646
rs9878436
rs7221585
rs10905255
rs6584579
rs142426306
rs56061761
rs116863223
rs4616688
rs7099229
rs1611236
rs11584821
rs8102497
rs80324517
rs10845387
rs142730696
rs11412296
rs1957937
rs6669563
rs28577594
rs6054257
rs61405042
rs77732866
rs9398196
rs10805346
rs3767952
rs34550383
rs7209057
rs144204502
rs139795227
rs2293579
rs1985369
rs56799554
rs869785
rs41269079
rs117034449
rs2977608
rs79977579
rs17445108
rs3785074
rs80116508
rs16978028
rs111527438
rs2230590
rs8105767
rs4724
rs6659669
rs73730598
rs4731541
rs77231040
rs137901416
rs9940099
rs11557154
rs1291143
rs1003322
rs932002
rs35640778
rs2967355
rs28363070
rs131797
rs9419958
rs6881568
rs7164950
rs611646
rs35446936
rs7772289
rs11117354
rs11699829
rs7705526
rs11991877
rs143190905
rs12925933
rs10774624
rs10768683
rs141214782
rs12932179
rs9955360
rs75664430
rs6007020
rs11769630
rs17677991
rs202034370
rs5742915
rs112394943
rs61748181
rs762679
rs139669835
rs2763979
rs66731853
rs1023767
rs7790856
rs185174247
rs182059586
rs188918174
rs117630647
rs145114957
rs4498805
rs45604339
rs6536702
rs3891167
rs4743037
rs76065543
rs28502153
rs939916
rs117407747
rs1907702
rs12451892
rs4695407
rs17803849
rs150150565
rs2056726
rs1332941
rs4530278
rs871134
rs9600019
rs10112752
rs59409453
rs78491606
rs2555104
rs6590343
rs117512405
rs76219171
rs6776756
rs2282764
rs376641875
rs76666449
rs3093888
rs10773176
rs2276182
rs10840270
rs13062095
rs11579626
rs11426156
rs41304832
rs12412214
rs11212631
rs6790988
rs6587577
rs55747751
rs73581419
rs429358
rs2538745
All − MR Egger
All − Inverse variance weighted
−2
−1
0
1
2
MR effect size for
'telomere length || id:ieu−b−4879' on 'IDP_dMRI_TBSS_OD_Tapetum_L || id:u
